# Lymphovascular Invasion and the Decision for Postmastectomy Radiation Therapy: A Cautionary Case Report

**DOI:** 10.1097/GOX.0000000000002115

**Published:** 2019-02-08

**Authors:** J. Arthur Jensen, Rania Bakkar, Michele Burnison, Armando E. Giuliano

**Affiliations:** From the * Division of Plastic Surgery, Cedars Sinai Medical Center, Los Angeles, Calif.; † Division of Pathology, Cedars Sinai Medical Center, Los Angeles, Calif.; ‡ Division of Radiation Therapy, Cedars Sinai Medical Center, Los Angeles, Calif.; § Division of Surgical Breast Oncology, Cedars Sinai Medical Center, Los Angeles, Calif.

## Abstract

Breast reconstruction is frequently adversely affected by postmastectomy radiation therapy. Some radiation therapists recommend postmastectomy radiation therapy based on the finding of lymphovascular invasion in the context of other findings. However, the diagnosis of lymphovascular invasion varies between pathologists and institutions. Sometimes special endothelial cell stains and outside opinions are necessary for the decision for postmastectomy radiation therapy. This case report illustrates the variation in the diagnosis of lymphovascular invasion. Plastic surgeons must remain current on the standard indications for postmastectomy radiation therapy and on the basic findings of lymphovascular invasion.

The modern treatment of breast cancer seeks to deliver maximally efficient oncologic control in the context of a near-natural esthetic outcome. In the setting of patients needing or choosing mastectomy, postmastectomy radiation therapy (PMRT) often conflicts with the goal of restoring a natural breast.^1,2^ However, in some cases, use of PMRT obviates the need to remove the nipple areolar complex, thus enhancing esthetic outcome.^3^

The generally negative impact of previous radiation therapy (RT) on reconstructive outcomes is well known. Patients with a history of RT have triple the chance of wound breakdown after reconstruction and quadruple the chance for expander or implant removal.^4^ Patients who receive PMRT also have elevated risk of implant or expander exposure and removal. Although patients who have autologous tissue breast reconstruction also experience increased surgical complications after RT,^5^ autologous tissue reconstruction is often required to salvage esthetic breast reconstruction in the setting of previous radiation or complications involving implants.

Mortality in breast cancer is almost always determined by progression to metastatic disease. LVI has been shown to predict adverse clinical outcomes—including nodal metastasis, local failure, and diminished overall survival. LVI is used by many oncologists to determine whether the patient would benefit from additional local (irradiation) or systemic treatment.

**Fig. 1.**
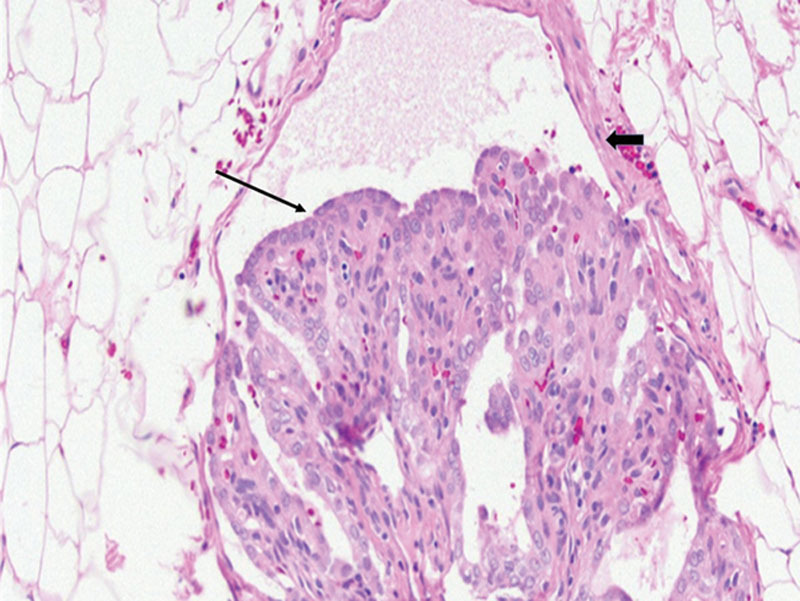
Hematoxylin and eosin section showing true lymphovascular invasion. Tumor embolus (arrow) is seen in a lymphovascular space lined by endothelial cells (arrow head). Note that the tumor embolus does not conform to the shape of the vascular space and that it is adherent to the vascular wall.

## CASE REPORT

**Fig. 2.**
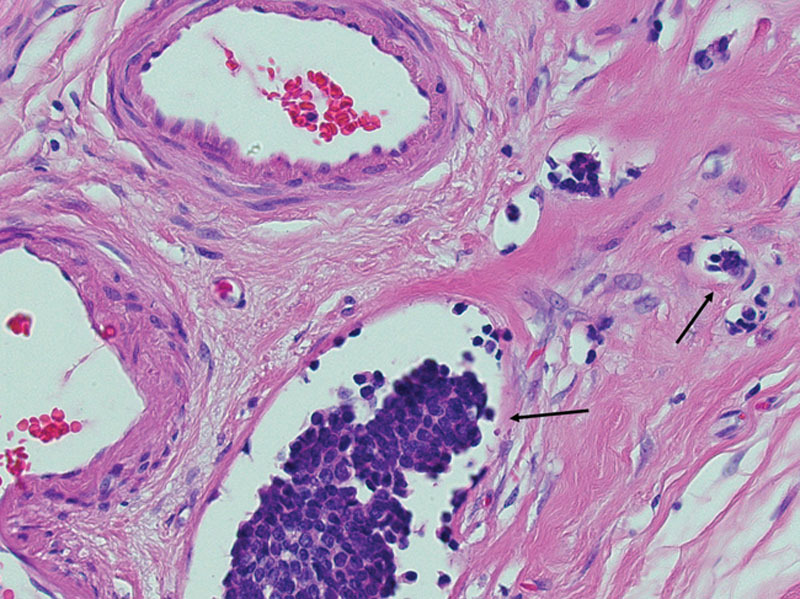
Hematoxylin and eosin stained histologic section from the breast specimen showing tumor cell clusters within spaces (arrows) associated with other thick walled vessels (upper left).

**Fig. 3.**
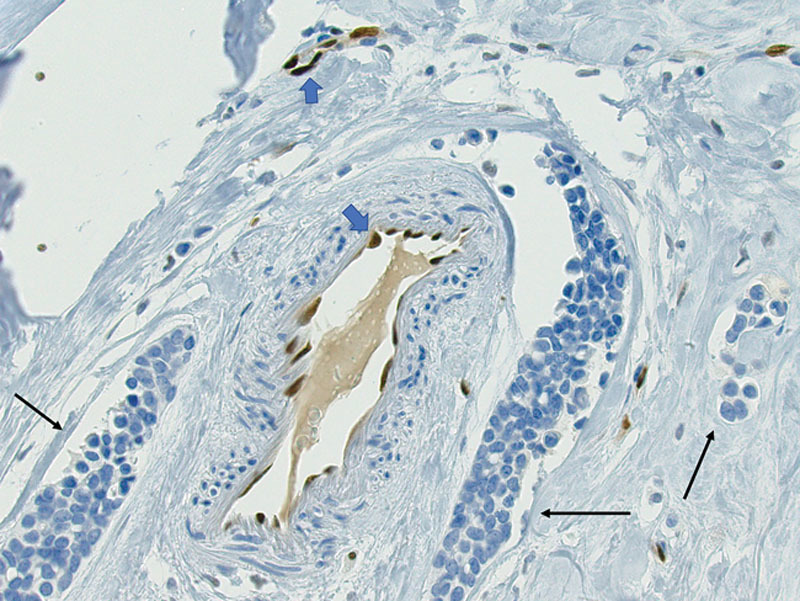
ERG endothelial immunohistochemical marker is negative in spaces containing tumor (arrows). Notice endothelial cells lining true vascular channels without tumor are positive for ERG (arrow heads).

**Fig. 4.**
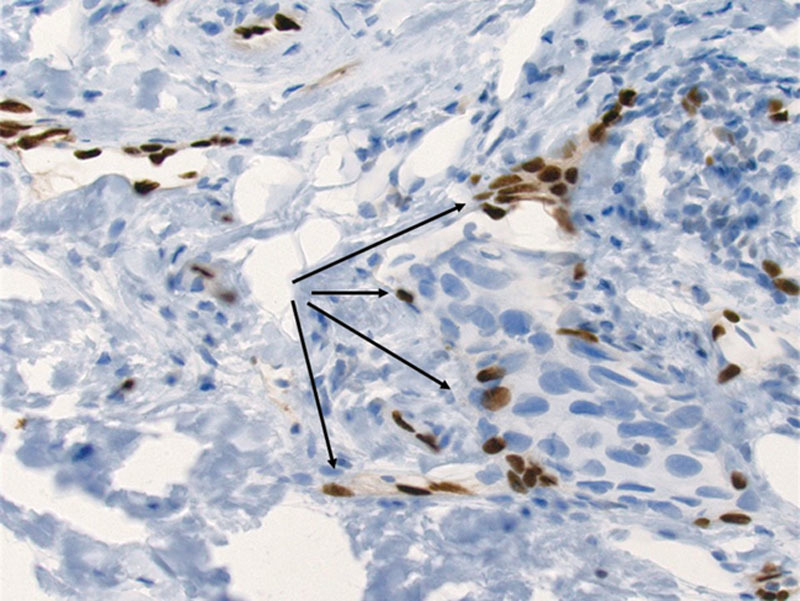
ERG is a nuclear stain that highlights endothelial cells (arrows). In this example, you can see nuclear positivity in endothelial cells lining a vascular space distended by a tumor embolus, supporting true LVI. Notice the strong degree of nuclear positivity that is easily visualized.

A 53-year-old woman noticed a skin retraction in her right breast and went for a mammogram. The mammogram was suspicious: an ultrasound and magnetic resonance imaging were followed by a core biopsy. The core biopsy rendered a diagnosis of multiple foci of invasive moderately differentiated ductal carcinoma, 1.4 cm in greatest dimension. The patient tested negative for BRCA (breast cancer) gene mutations. She received herceptin and 6 cycles of chemotherapy followed by bilateral skin and nipple-sparing mastectomies with immediate reconstruction with tissue expanders. Pathology of the breast showed residual invasive ductal carcinoma, 2 foci, 0.9 cm in greatest dimension, with probable/definite response to presurgical neoadjuvant therapy in the invasive component. Intramammary LVI was reported as positive and multifocal on routine hematoxylin and eosin stained histologic sections (Fig. 2). Three sentinel lymph nodes were removed from the axilla, one was positive for isolated tumor cells only by pancytokeratins immunohistochemistry. All margins, including subareolar margin, were widely negative for tumor. The pathologic stage was reported as ypT1b N0(i+) (sn). The case was reviewed at the Breast Multi-Disciplinary Tumor Conference. Based on the finding of multifocal intramammary LVI, PMRT was considered given the increased risk of local recurrence associated with such finding. Pathology rereviewed the histologic slides to determine the extent of LVI. However, the presence of LVI became questionable upon pathology intrainstitutional peer review. Ancillary studies including ERG and D2-40 (endothelial immunohistochemical markers) were performed on areas with questionable LVI to further verify this finding. The cells lining questionable lymphovascular spaces containing tumor were negative (Fig. 3); meaning that these spaces were not lined by endothelial cells, and therefore may represent stromal retraction artifact lined by fibroblasts around tumor cells rather than true LVI (Fig. 4). The histologic and immunohistochemical slides were sent out to other prominent pathology departments nationwide for additional consultation. The first outside institution agreed with the initial report of positive LVI based on the presence of the tumor-containing spaces in the right anatomic location (accompanying other big vessels and nerve bundles), whereas the second institution reported absence of LVI based on the endothelial markers that failed to highlight endothelial cells lining the tumor-containing spaces, favoring retraction artifact. The case was presented again at the Breast Multi-Disciplinary Conference, and the decision was made to not advise PMRT. The patient has subsequently completed her implant-based breast reconstruction and is followed without evidence of recurrent breast cancer or capsular contracture of the implant.

## DISCUSSION

Although the diagnosis of LVI is made by a pathologist, plastic surgeons should be aware of the basic diagnostic features of this finding and possible pitfalls of its use. Pathologists identify LVI when they see tumor emboli in the lymph or vascular structures distant from the tumor.^6–9^ The diagnosis of LVI cannot be made within the substance of the tumor. Emphasis is also placed on the identification of endothelial cells lining the lymphovascular spaces wherein the tumor emboli are located. Tumor emboli should be adherent to the vascular endothelium and most often do not conform exactly to the shape of the lymphovascular space. Sometimes tumor cells mimic LVI by appearing as knife carryover artifact which might exactly conform to the shape of the vessel. Stromal retraction artifact around tumor cells with fibroblasts mimicking endothelial cells on routine studies is the most common source of discrepancy in the diagnosis of LVI.

The utility of this diagnostic finding is limited by interobserver variability. This may be due to variations in the threshold for diagnosis and whether ancillary histochemical studies are used to aid in diagnosis. The presented case illustrates difference of opinion in the same institution and in other internationally prominent institutions.

Differences of opinion regarding the diagnosis of LVI are critical to the management of certain patients. A broad review^10^ of the indications for PMRT outlines areas of basic agreement for RT treatment and nontreatment: patients with inflammatory cancers should be treated; patients with ≥4 positive lymph nodes and/or cancers >5 cm generally should be treated; patients with negative nodes generally should not be treated; and patients with 1–3 positive nodes and LVI should be thoroughly discussed in a tumor board setting and decisions scrutinized. Because PMRT may result in significant emotional and financial costs to patients, every effort should be made to confirm this finding by additional ancillary studies and/or obtain pathology consensus before final recommendations are made. Efforts should be made by the pathology community to standardize the use of ancillary endothelial markers to support this diagnosis.

As members of multidisciplinary tumor boards, plastic surgeons may contribute insights into the impact of PMRT on outcomes of breast reconstruction. Understanding the features and controversies of LVI facilitates this important interaction.
